# An unexpected variation of occipital and ascending pharyngeal arteries: clinical implications

**DOI:** 10.1007/s00276-024-03511-w

**Published:** 2024-11-28

**Authors:** Gabriella Roofeh, Nicholas Matthees, Manuel Cevallos

**Affiliations:** 1 Creighton University School of Medicine—Phoenix Regional Campus, Phoenix, AZ USA; 2 Department of Radiology Phoenix, Mayo Clinic Arizona, AZ, USA

**Keywords:** Ascending pharyngeal artery, Occipital artery, Internal carotid artery, Variant, Carotid endarterectomy, CT angiogram

## Abstract

**Purpose:**

The eight typical External Carotid Artery (ECA) branches are the superior thyroid artery, ascending pharyngeal artery, lingual artery, facial artery, occipital artery, posterior auricular artery, maxillary artery, and superficial temporal artery. The Internal Carotid Artery (ICA) has no branches in the cervical region before entering the carotid canal. We identified a variant of the Occipital Artery (OA) and Ascending Pharyngeal Artery (APA) originating in the cervical portion of the ICA and wish to explore the clinical implications this variation may have.

**Methods:**

Between August and December 2023, 28 formaldehyde donors were dissected in the anatomy course for first-year medical and physician assistant students at the Creighton University School of Medicine, Phoenix campus.

**Results:**

In one donor, dissection of the right common carotid artery (CCA) revealed a variation of the ECA branches. Two branches were found on the proximal portion of the ICA. After tracing the branches cranially, we identified these as the OA and APA. These two arteries typically originate from the ECA. The bifurcation angle was observed to be nearly 180 degrees. This variation was only observed on the right side.

**Conclusion:**

As the prevalence of this variation has only been described in one study, reporting at 0.14%, documentation and education of this anatomy aids surgeons and interventional radiologists in head and neck procedures. Exploring the paths of these ectopic arteries promotes informed decision making and risk stratification for carotid endarterectomy, arterial embolization, bypass procedures, and arterial harvesting. Furthermore, performing thorough imaging such as CT angiograms on patients preoperatively provides better foresight to minimize complications.

## Introduction

In an age of rapidly advancing technology, imaging, and surgical techniques, additional treatment options have become available for diverse pathologies that previously required more extensive or invasive interventions. Due to the preference and ability to offer minimally invasive interventions for various procedures of the head and neck, surgeons and interventional radiologists would significantly benefit from a comprehensive understanding of anatomical structures and variations present in the population. We aim to describe a variation in ectopic carotid artery branches, their prevalence, embryologic origins, and the clinical implications of anomalies like the one identified in this cadaveric donor, with independent aberrant origins of the occipital artery and ascending pharyngeal artery from the internal carotid artery.

As the Common Carotid Artery (CCA) ascends through the neck, it bifurcates at the level of the superior border of the thyroid cartilage. This branching gives rise to the internal carotid artery and the external carotid artery. The eight External Carotid Artery (ECA) branches are the superior thyroid artery, ascending pharyngeal artery, lingual artery, facial artery, occipital artery, posterior auricular artery, maxillary artery, and superficial temporal artery. Each branch takes a unique course through the head to supply its designated region and is expected to develop symmetrically. The extracranial Internal Carotid Artery (ICA) typically has no branches in the cervical portion before entering the carotid canal. We identify a variant of both the Occipital Artery (OA) and Ascending Pharyngeal Artery (APA) originating from the cervical portion of the ICA.

With two variant branches, each of their unique paths demonstrates clinical relevance and requires extensive knowledge to manipulate. The OA’s typical orientation presents as the fifth branch originating from the ECA, coursing superficially to the ICA posteriorly towards the occipital region of the head [1]. As it courses posteriorly, it supplies head and neck muscles including the sternocleidomastoid and splenius capitis [6]. As it approaches the superior nuchal line of the occipital bone, it turns vertically and continues up the back of the head. There, it contributes perfusion to the superior portion of the trapezius muscle, the semispinalis capitis, and even as anteriorly as the occipitofrontalis muscle. Its proximity to vessels like the posterior inferior cerebellar artery (PICA) allows it to serve as a conduit for bypass in cases of occlusion or aneurysm. The OA’s typical configuration also provides potential for collateral circulation to the vertebral artery in cases of ICA occlusion.

The path and arterial territory of the APA lie more anteriorly. It typically branches from the posterior border of the ECA and immediately travels vertically [7]. As it ascends parallel to the ICA, it divides into numerous vessels which further course towards their respective regions. These include the middle pharyngeal artery, superior pharyngeal artery, inferior tympanic artery, hypoglossal branch, jugular branch, musculospinal branch, and odontoid arch system. These distinct paths allow the APA to span its perfusion from the ear to the pharynx, soft palate, and further to the meninges and even cranial nerves IX, X, XI, and XII. A recent report depicts a variation of the APA, in which its jugular branch continues to become the PICA [16]. This was in combination with the PICA that is expected to arise from the vertebral artery, which resulted in a duplicated PICA on the left side, and a normally originating individual PICA on the right side.

### Case report

Between August and December 2023, 28 donors preserved at 0.1% formaldehyde were dissected in the anatomy course for first-year medical and physician assistant students at the Creighton University School of Medicine, Phoenix Regional Campus. In an 88-year-old female donor, whose cause of death was documented as dementia due to Parkinson’s disease, dissection of the right CCA revealed a variation of the ECA branches. On this side, the CCA bifurcated at a nearly 180-degree angle at the level of the hyoid bone. We identified this corresponding to the C3 vertebral level.

**Fig. 1:**
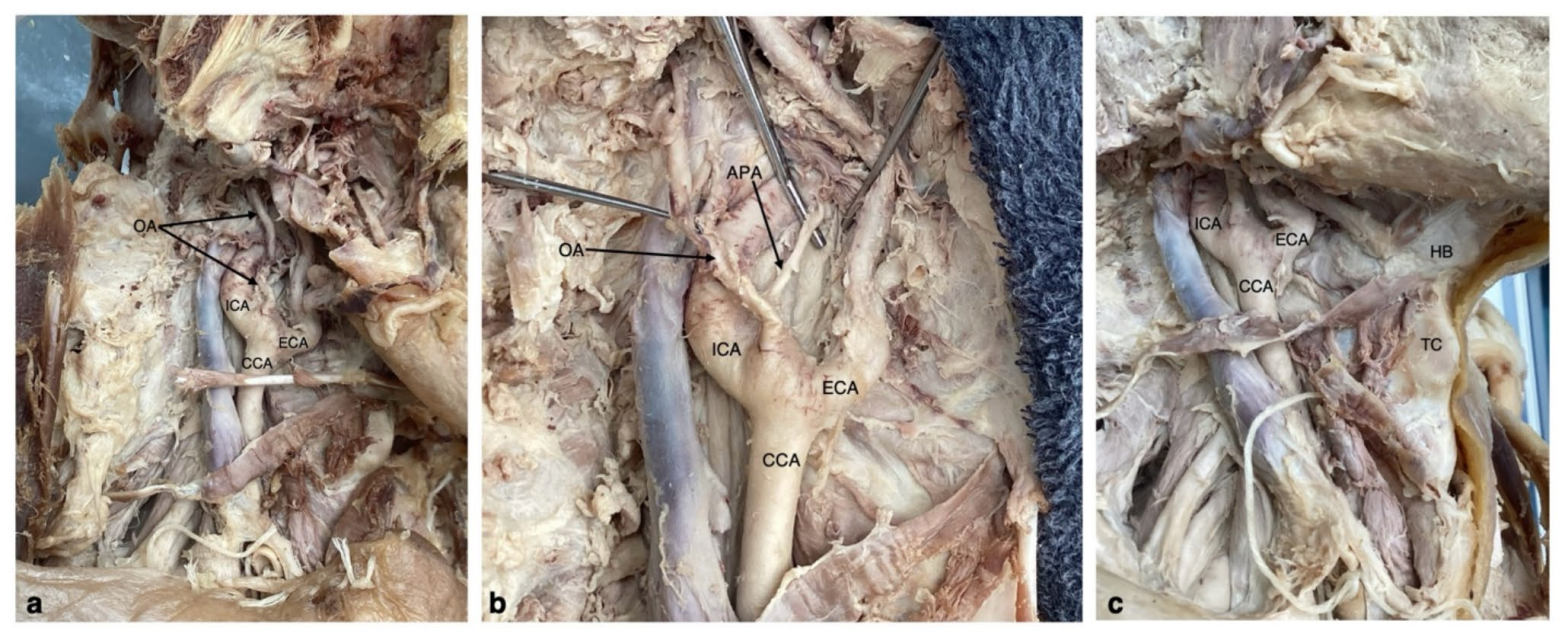
(**a**) Original position of the right common carotid artery (CCA) discovered in dissection, with a view of the internal carotid artery (ICA), external carotid artery, and an aberrant occipital artery (OA) originating at the proximal segment of the ICA. (**b**) Dissection of the right sided carotid sheath with exposure of the carotid bifurcation reveals the internal carotid artery (ICA) ascending posterolaterally while the external carotid artery (ECA) ascends anteromedially. On the proximal ICA, two branches are identified: the occipital artery (OA) on the lateral side and the ascending pharyngeal artery (APA) on the medial side. (**c**) Dissected right CCA bifurcation and the anterior neck depict the carotid bifurcation at the level of the Hyoid Bone (HB). CCA- Common Carotid Artery, ECA- External Carotid artery, ICA- Internal Carotid Artery, OA- Occipital Artery, APA- Ascending Pharyngeal Artery, HB- Hyoid Bone, TC- Thyroid Cartilage

**Fig. 2:**
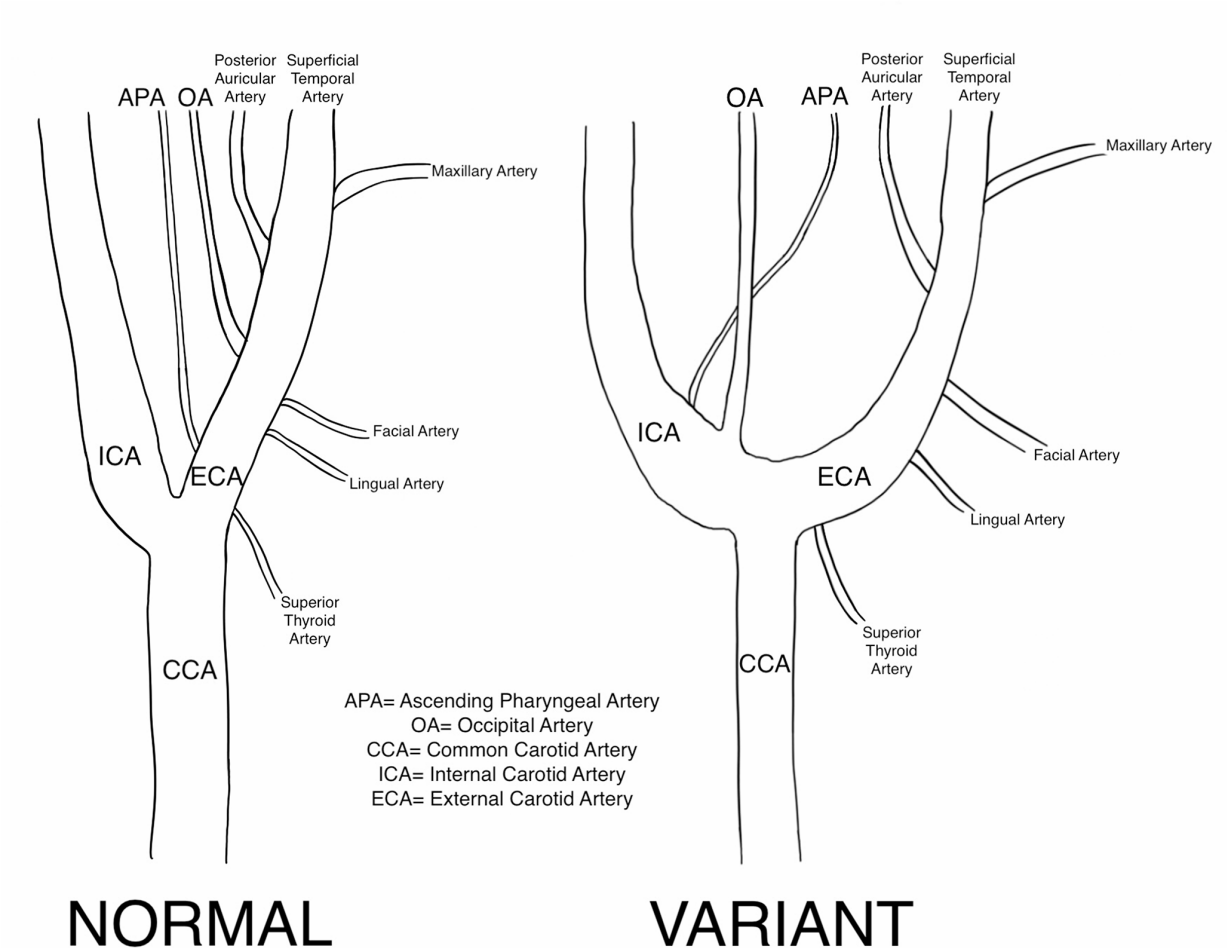
Diagram depicting the branches of the right carotid artery in the typical form, with eight branches originating on the ECA, and the variant discovered, with the OA and APA originating on the ICA and the remaining 6 branches originating on the ECA

The ECA traveled anteromedially, and the ICA coursed posterolaterally, as we expected. Just distal to the point of bifurcation, an artery was identified branching off the lateral portion of the ICA, which we identified as the OA. Additional dissection also revealed an aberrant APA originating on the medial aspect of the proximal ICA (Fig. 1). Due to the nature of the wide angle, the aberrant arteries identified on the dilated carotid sinus portion of the proximal ICA were found branching at the C3 vertebral level. This is in contrast to recent cases of aberrant OA origins on the ICA detected by MR angiography occurring at the level of the C2 vertebra [15, 17]. These two arteries typically originate from the ECA (Fig. 2). The remaining six ECA branches were identified in their typical configurations. This variation was only observed on the right side. The left CCA bifurcated as expected at the level of the superior border of the thyroid cartilage.

A study performed by Saho and Onishi found that the bifurcation angle has a negative correlation with wall shear stress due to reduced energy of blood flow. This was associated with an increased risk for the development of atherosclerosis [13]. In the case presented, palpation revealed that these vessels were soft and malleable. The artery walls displayed adequate compliance, and we did not identify any firmness or nodules suggesting no palpable evidence of atherosclerotic plaques. Dissection into the artery was not performed as preservation of this structure was a limitation to maintaining the viability of use for academic courses. Measurements of the distance of these arteries from the carotid bifurcation were not obtained.

## Discussion

### Embryology

Development of the great vessels of the head and neck including the carotid arteries begins with the truncus arteriosus [10]. This is the most cranial portion of the primitive heart after folding during embryologic development. The aortic sac is the most distal portion of the truncus arteriosus and forms in week three of gestation. From the aortic sac, the aortic arches begin forming in the fourth week, one for each pharyngeal arch. These six arches develop and regress at different times and are numbered chronologically. The remnant of the first aortic arch is the maxillary artery, a distal branch of the ECA. From the second aortic arch, we get the hyoid artery, a distant branch of the superficial temporal branch from the ECA, and the stapedial artery, which eventually regresses as well but provides collateral circulation during development between the ICA and ECA branches. The third aortic arch gives rise to the CCA and proximal portions of the ICA bilaterally; its distinct fate warrants its colloquial name, the “carotid arch”. Arches four, five, and six do not contribute to the carotid arteries.

Between weeks six and eight of embryologic development, these vessels undergo further transformation into the basic human adult arteria. The additional branches of the ECA have complex developmental patterns which have been debated thoroughly. The embryological origin of this OA variation is unclear but has been hypothesized by Lasjaunias et al. to be a type 1 remnant of the proatlantal intersegmental artery [19]. This embryonic vessel typically provides an anastomosis between the ICA and vertebral artery, regressing before birth. Incomplete regression of this branch may result in an OA arising from the ICA. Another theory of an aberrant OA origin on the ICA is a type 2 remnant of the proatlantal artery, with regression of its most proximal segment and of the distal anastomosis from the vertebral artery [17]. Demirbas et al. report a case of bilateral OA originating from the vertebral artery, displaying characteristics of a proatlantal artery remnant [3]. During the development of the APA, the artery initially grows on the ICA, and in later stages regresses and shifts to the ECA. Lasjaunias et al. predict that an ectopic origin of the APA on the ICA is a result of a halt in the artery’s development during such stages of regression and regrowth [19].

### Clinical Implications

The prevalence of aberrant ECA branches originating on the ICA has been well described and documented. Lippert and Pabst, in 1985, reported the OA originating on the ICA to occur in less than 0.1% of individuals, while an APA originating on the ICA is more common and is found in 8% of individuals [5]. Later, in 2012, Iwai et al. identified 1 OA originating on the ICA out of the 265 patients by analysis of CT angiography [9]. In a retrospective analysis of MR angiography on 2,866 patients, Uchino et al. found that 5 OA originated on the ICA, and 1 was found originating on the anterior aspect of the carotid bifurcation, suggesting an incidence of 0.21% for anomalous OA origins [18]. In this study, the authors were unable to detect APA origins, attributed to the low spatial resolution of 1.5T MR angiogram. In a 5 year retrospective review by Small et al., researchers investigated the extracranial branches of the ICA by analyzing 5,064 CT angiograms. They reported a 6.25% prevalence of APA arising from the ICA, which is notably more common than that previously discussed regarding the OA. Additionally, they reported that 0.14% (*n* = 7) of the 5,064 arteries analyzed had independent OA and APA origins on the ICA, determining that the combination of these variations occurring in one patient is an uncommon finding [14]. Due to the rarity of such variations, studying the anatomy and understanding the paths of ectopic arteries bears great clinical significance to support surgeons and interventional radiologists. Awareness of variations, assessing patients’ candidacy for procedures, and considering alternative treatment plans are essential in providing informed patient care.

Understanding the anatomy and doing thorough imaging in advance is essential in procedures like carotid endarterectomy (CEA). This is performed on patients to remove plaque buildup in the carotid arteries. It has become increasingly more common with the rise in atherosclerotic disease. For symptomatic carotid artery stenosis patients experiencing TIAs, stroke, or asymptomatic patients with greater than 70% narrowing, this procedure is considered for improvement [20]. In CEA, duplex ultrasonography is typically the preoperative imaging of choice due to accessibility, cost, and its non-invasive nature; however, there is relatively little anatomic detail, and such variations may be missed. Furthermore, in a procedure with a small dissection field, a cervical ICA with no branches helps surgeons distinguish it from the ECA; discovering ectopic ICA branches during surgery may delay protocol and call for additional imaging and resources before proceeding. Damage to these vessels, when discovered during surgery, can compromise circulation to the regions they supply.

Hemodynamic instability is another concern during CEA and other procedures with close contact with the carotid sinus. There are currently no studies with evidence that anesthetics can prevent such fluctuations in blood pressure intra and post-operatively, but many surgeons still practice this method as a precaution [20]. Carotid sinus stimulation during intervention predisposes a patient to coronary vasospasm and resultant myocardial infarction [1]. In a procedure like OA-PICA bypass in cases of a PICA aneurysm, the OA is resected from its origin [4]. Resecting a branch from the ICA introduces a risk of cerebral infarction or compromise to the carotid sinus considering its proximity.

Additional complications of intervention include back bleeding and embolic events. Back bleeding occurs when branches like the APA originate between the incision site and the proximal point of clamping, filling the surgical field with blood [19]. In CEA, plaques within these branches near the bifurcation or with ICA origins can become dislodged and cause embolic stroke. Heparin is typically administered for such procedures, and intraoperative surgical techniques like clamping to localize the field and avoid clots from traveling are also in place to prevent embolic events [20]. In addition to all the measures currently in place, performing preoperative CT angiograms would help identify any anatomical variations [14].

Symptomatic presentation of the OA arising from the ICA has been documented to cause pulsatile tinnitus [2]. Carotid stenosis and subsequent reversal of flow and turbulence through the ectopically placed artery were the suggested reasons for such symptoms, which resolved with stent placement at the site of stenosis. When performing carotid stenting procedures, securing these branches is pertinent. Protection devices should be used to ensure these arteries are not damaged or embolized [8]. Once ectopic origins of branches are identified on the ICA with preoperative imaging, they should be protected accordingly to minimize risks.

Aberrant arteries inherently have poor integrity. Each branching point of an artery poses a risk for aneurysm, and for variations like the one we found, rupture can compromise cerebral circulation due to ICA hemorrhaging, as well as anterior and posterior circulation provided for by these smaller arteries. Keshelava et al. report a case of cervical ICA aneurysm, and intervention revealed a carotid bifurcation angle of 180 degrees, with an APA originating at the bifurcation [11]. Blood stasis in stable aneurysms presents yet another risk for cerebral infarction in these locations.

Another intervention with a considerable increase in risk due to ectopic artery origins is catheterization or embolization. The OA and APA have both been used safely and successfully for chemotherapy administration for late-stage head and neck cancers. Still, they may need to be avoided in cases of ectopic origins [8, 12]. The occipital artery can be used for superselective intraarterial chemotherapy for late-stage oral cancers, like stage III or IV oral squamous cell carcinoma [8]. In this technique, the OA is used as the insertion point for microcatheterization. The superficial temporal artery is commonly used for such treatment, often concurrently with the occipital artery. If the OA arises from the ICA, this procedure introduces a risk of cerebral infarction and should not be used. In these cases, Mitsudo et al. suggest that surgical resection should be considered, or use of solely the superficial temporal artery or femoral artery can be used as alternatives if they are available [12]. Important attention and care should be used in cases of transarterial embolization of the OA if its origin is on the ICA [6]. In addition to its use for superselective intra-arterial chemoradiotherapy combinations for nasopharyngeal carcinoma, the APA has been successfully used as a microcatheterization site for palatal cancer embolization [7]. This procedure also poses a threat of cerebral infarction if the APA has an origin on the ICA and should be analyzed before performing.

## Conclusion

Procedures of the head and neck require significant knowledge of the anatomy of the carotid arteries and their branches, including the OA and APA. Having ectopic ECA branches originating on the ICA and wide bifurcation angles may lead to weakened vasculature and additional risks for CEA and other procedures. Documenting and describing variations in vasculature amongst the population supports surgeons and interventional radiologists. Unidentified variations in head and neck vasculature may delay treatment when undetected by ultrasonography. Incorporating imaging such as CT angiogram, although more expensive, can aid preoperative treatment planning with its increased spatial resolution and ability to detect anomalous arteries.

## Data Availability

No datasets were generated or analysed during the current study.
